# Development of Colloidal Gold Immunochromatographic Strips for Detection of *Riemerella anatipestifer*

**DOI:** 10.1371/journal.pone.0122952

**Published:** 2015-03-30

**Authors:** Wanwan Hou, Shaohui Wang, Xiaolan Wang, Xiangan Han, Hongjie Fan, Shoulin Cao, Jiaping Yue, Quan Wang, Wei Jiang, Chan Ding, Shengqing Yu

**Affiliations:** 1 Shanghai Veterinary Research Institute, Chinese Academy of Agricultural Sciences, Shanghai 200241, China; 2 Key Lab of Animal Bacteriology, Ministry of Agriculture, Nanjing Agricultural University, Nanjing 210095, China; Queen's University at Kingston, CANADA

## Abstract

*Riemerella anatipestifer* is one of the most important bacterial pathogen of ducks and causes a contagious septicemia. *R*. *anatipestifer* infection causes serositis syndromes similar to other bacterial infections in ducks, including infection by *Escherichia coli*, *Salmonella enterica* and *Pasteurella multocida*. Clinically differentiating *R*. *anatipestifer* infections from other bacterial pathogen infections is usually difficult. In this study, MAb 1G2F10, a monoclonal antibody against *R*. *anatipestifer* GroEL, was used to develop a colloidal gold immunochromatographic strip. Colloidal gold particles were prepared by chemical synthesis to an average diameter of 20±5.26 nm by transmission electron microscope imaging. MAb 1G2F10 was conjugated to colloidal gold particles and the formation of antibody-colloidal gold conjugates was monitored by UV/Vis spectroscopy. Immunochromatographic strips were assembled in regular sequence through different accessories sticked on PVC plate. Strips specifically detected *R*. *anatipestifer* within 10 min, but did not detect *E*. *coli*, *S*. *enterica* and *P*. *multocida*. The detection limit for *R*. *anatipestifer* was 1×10^6^ colony forming units, which was 500 times higher than a conventional agglutination test. Accuracy was 100% match to multiplex PCR. Assay stability and reproducibility were excellent after storage at 4°C for 6 months. The immunochromatographic strips prepared in this study offer a specific, sensitive, and rapid detection method for *R*. *anatipestifer*, which is of great importance for the prevention and control of *R*. *anatipestifer* infections.

## Introduction

*Riemerella anatipestifer* is a Gram-negative, non-motile, rod-shaped bacterium that is one of the most important bacterial pathogen of ducks. *R*. *anatipestifer* infection causes a contagious septicemia characterized by fibrinous pericarditis, airsacculitis, perihepatitis, and caseous salpingitis. At least 21 serotypes of *R*. *anatipestifer* have been identified [1–3], with serotypes 1, 2, and 10 are the most prevalent in China [4]. *R*. *anatipestifer* infection causes serositis syndromes similar to other bacterial infections in ducks, including infection by *Escherichia coli*, *Salmonella enterica* and *Pasteurella multocida*. Clinically differentiating *R*. *anatipestifer* from these bacterial pathogen infections is difficult.

Laboratory methods available for detecting *R*. *anatipestifer* include serological methods such as agglutination test, agar gel immunodiffusion test (AGID), and enzyme-linked immunosorbent assay (ELISA) [5], which are the most commonly used methods. Agglutination tests and AGIDs identify *R*. *anatipestifer* serotypes [6], while ELISAs use antigens to detect *R*. *anatipestifer* antibody [5]. Recent molecular biological methods include polymerase chain reaction (PCR) [7], multiplex PCR (m-PCR) [8] assay, and loop-mediated isothermal amplification (LAMP) [9] assays; however, these procedures are very cumbersome since they require skilled technicians, special equipments and reagents, and several hours to perform. Therefore, an efficient, rapid, specific, and easily performed method for detection of *R*. *anatipestifer* is critically needed.

Colloidal gold immunochromatographic strips are new, rapid, single-step immunochromatographic assays [10] that use colloidal gold as the tracer. Since Beggs and Osikowicz first developed the colloidal gold immunochromatography assay for qualitative detection of human chorionic gonadotropin (HCG) [11], this type of assay has been widely applied to diagnose many diseases. Its advantages are its rapid, simple, specific and sensitive characteristics. In addition, the method has been used to detect bioactive molecules, hormones, and haptens [12–13]. In detection of microorganisms of veterinary importance, colloidal gold immunochromatographic strips have been used to detect bovine virus diarrhea and white spot syndrome viruses [14–15]. However, the assay has not been reported for the detection of *R*. *anatipestifer*. We generated colloidal gold immunochromatography strips for detecting *R*. *anatipestifer* using a monoclonal antibody (MAb) against *R*. *anatipestifer* GroEL protein. The colloidal gold immunochromatographic strips we developed offer a method for specifically, sensitively, and rapidly detecting *R*. *anatipestifer*.

## Materials and Methods

### Bacterial strains, growth conditions and chemical reagents

Bacterial strains used in this study and their sources are in Table 1. Strain WJ4 was deposited in the China General Microbiological Culture Collection Center (CGMCC no. 5264) and chosen for developing specific, sensitive and stable colloidal gold immunochromatographic strips. *R*. *anatipestifer* strains were cultured at 37°C in tryptic soy broth (TSB, Difco, Detroit, MI, USA). *E*. *coli* and *S*. *enterica* strains were cultured at 37°C in Luria broth (LB, Difco). *P*. *multocida* strain CVCC 493 was grown aerobically at 37°C in brain heart infusion medium (BHI, Difco).

**Table 1:** Bacterial strains used in this study.

Strains	Description	Source or reference
WJ4 (CGMCC 5264)	*R*. *anatipestifer* strains, serotype 1	CGMCC^a^
CH3, CH1, CQ1, CQ3, CQ4, CQ5, JY4, YXb12, NN2, NJ1, NJ4, YL4	*R*. *anatipestifer* strains, serotype 1	[16]
JY1, SC2, NJ3, Yb2, Th4, YXb1, NN1, NN5, GD3, GD4, GD5	*R*. *anatipestifer* strains, serotype 2	[16]
P2123	*R*. *anatipestifer* strains, serotype 6	NADC^b^
D26220	*R*. *anatipestifer* strains, serotype 8	DRL^c^
YXb11, HXb2, YXL1	*R*. *anatipestifer* strains, serotype 10	[16]
8785	*R*. *anatipestifer* strains, serotype 12	CCUG^d^
D743	*R*. *anatipestifer* strains, serotype 15	CVL^e^
NN6	*R*. *anatipestifer* strains, serotype 15	[16]
JY6, GD6	*R*. *anatipestifer* strains, serotype ND^f^	[16]
CVCC1547	APEC reference strain	CVCC^g^
APEC O1	APEC strain, serotype O1	[17]
DE171	APEC strain, serotype O1	[18]
DE14	APEC strain, serotype O2	[18]
DE17	APEC strain, serotype O2	[18]
CE66	APEC strain, serotype O18	[18]
DE48	APEC strain, serotype O78	[18]
DE56	APEC strain, serotype O78	[18]
CVCC 1805	*S*. *enterica serovar enteritidis*	CVCC
CAU 0118	*S*. *enterica serovar anatum*	CVCC
JXb1	*S*. *enterica serovar typhimurium*	[8]
CVCC 3384	*S*. *enterica serovar typhimurium*	CVCC
CVCC 519	*S*. *enterica serovar pullorum*	CVCC
CVCC 493	*Pasteurella multocida*	CVCC

^a^CGMCC, China General Microbiological Culture Collection Center, China

^b^NADC, National Animal Disease Center, Ames, Iowa, USA

^c^DRL, Duck Research Laboratory, New York, USA

^d^CCUG, Culture Collection, University of Goteborg, Sweden

^e^CVL, Central Veterinary Laboratory, Singapore

^f^ND, not determined

^g^CVCC, Chinese Veterinary Culture Collection Center, China.

Chemical reagents including gold chloride (HAuCl_4_﹒3H_2_O), sodium citrate (C_6_H_5_Na_3_O_7_﹒2H_2_O), sodium azide, Tween 20 and polyvinylpyrrolidone K30 were from Sigma (St. Louis, MO, USA).

### Recombinant *R*. *anatipestifer* GroEL protein and anti-GroEL MAb

Recombinant *R*. *anatipestifer* GroEL protein (rGroEL) and anti-GroEL MAb were prepared in our laboratory previously [19]. Briefly, the *groEL* gene was cloned from *R*. *anatipestifer* strain WJ4 and rGroEL was expressed and used as an immunization antigen. BALB/c mice were immunized for 3 times, and the hybridoma technique was performed for the MAb development. Positive clones were screened using indirect ELISA and sub-cloned three times. The hybridoma cells producing anti-GroEL MAb 1G2F10 were obtained and deposited in China General Microbiological Culture Collection Center (CGMCC no. 8778). Anti-GroEL MAb 1G2F10 was used to develop the colloidal gold immunochromatographic strips. Ascites fluid titers of 1G2F10 were higher than 1:102,400 by ELISA. By Western blot, 1G2F10 reacts well with *R*. *anatipestifer* serotypes 1, 2, and 10 strains, but not react with *E*. *coli*, *S*. *enterica*, and *P*. *multocida* strains [19]. MAb was purified from ascites fluid using affinity chromatography and characterized by SDS-PAGE.

### Preparation and characterization of colloidal gold

Colloidal gold particles with a mean diameter of 20 nm were prepared according to the method of Frens [20]. Briefly, under rapid magnetic stirring, 100 mL of 1% (w/v) chloroauric acid was boiled thoroughly, and then 1.7 mL 1% (w/v) trisodium citrate solution was added. The color changed to wine red in 3 min and the colloidal gold solution was boiled for 10 min and gradually cooled. Colloidal gold solution can be stored at 4°C for a few months. Colloidal gold particles were characterized by transmission electron microscopy (Tecnai G2, FEI, Netherlands).

### Preparation of colloidal gold-MAb conjugate

MAb 1G2F10 (0.03 mL, 1 mg/mL) was added to 10 mL of colloidal gold solution, and stirred vigorously for 30 min. Bovine serum albumin (BSA) aqueous solution (5% w/v, 2.5 mL) was added to block excess colloidal gold reactivity. After stirring for 30 min, the mixture was centrifuged at 12,000 rpm, 4°C for 20 min to remove unbound MAb. The resulting conjugate pellet was resuspended in 1 mL of 0.01 M Tris-HCl buffer (pH 8.0). Conjugation of colloidal gold to antibodies was examined by UV/Vis spectroscopy (Thermo) as described [21].

### Preparation of colloidal gold immunochromatographic strips

Colloidal gold immunochromatographic strips had four components, a sample pad, a conjugate pad, a nitrocellulose membrane and an absorbent pad. The sample pad (JieYi Biotech, Shanghai, China) was saturated with 0.01 M phosphate buffered saline (PBS) solution (pH 7.4) containing 0.2% Tween-20 and dried at 37°C for 2 h and stored in a desiccator at 4°C.

The conjugate pad (glass fiber membrane, catalog no. CN95, Sartorius, JieYi Biotech) was treated with blocking solution of 0.01 M PBS containing 2% BSA, 0.5% sucrose, 0.5% Tween-20, 0.5% polyvinylpyrrolidone K30, and 0.02% sodium azide (pH 7.4) and dried at 37°C before use. Colloidal gold probe was jetted onto the glass fiber and dried at 37°C for 2 h and stored in a desiccator at 4°C.

MAb 1G2F10 (1 mg/mL) and the goat anti-mouse antibody (MAI Bio Co. Ltd. Shanghai, China, 1 mg/mL) in PBS were dispensed onto the nitrocellulose membrane (Millipore, JieYi Biotech) in two discrete zones: one for test line (T line) and the other for control line (C line). An XYZ platform (BioDot, USA) was used with a volume of 5 μL/cm. The nitrocellulose membrane was dried at 37°C for 2 h and stored in a desiccator at 4°C.

A PVC plate (JieYi Biotech) was used as the base of the test strip. The absorption pad, nitrocellulose membrane, conjugate pad, and sample pad were attached sequentially to the PVC plate with a 1 to 2 mm overlap. A CM 4000 cutter (BioDot) was used to cut the assembled plate into 3 mm-wide pieces. Strips were stored in a desiccator at 4°C.

### Specificity, sensitivity and stability of colloidal gold immunochromatographic strip

*R*. *anatipestifer* strains of different serotype and samples containing other antigens were used to evaluate the specificity of colloidal gold immunochromatographic strip. Samples were diluted to 1×10^8^ colony forming units (CFU)/mL with 0.01 M PBS solution (pH 7.4) and 100 μL of sample was added to the sample pad. Samples were also detected by m-PCR and conventional agglutination tests to evaluate the accuracy of the colloidal gold immunochromatographic strips. The m-PCR was performed using primers RA DnaB P1 (AAACTCAGGCAAAGGTGGCAC) and RA DnaB P2 (TGTATGGTAGTTTTGATGCTTTCAA) as described previously [8]; the gene product was 459 bp.

The sensitivity of colloidal gold immunochromatographic strips was evaluated by *R*. *anatipestifer* strain WJ4 at different densities (1 × 10^9^, 1 × 10^8^, 1 × 10^7^, 1 × 10^6^, or 1 × 10^5^ CFU/mL), prepared by 10-fold dilutions with 0.01 M PBS solution (pH 7.4). Dilution (100 μL) was added to the sample pad and 0.01 M PBS solution (pH 7.4) was used as the blank control. The same samples were also used for m-PCR and conventional agglutination tests for sensitivity test.

Colloidal gold immunochromatographic strips were stored at 4°C for 6 months to evaluate stability during storage. *R*. *anatipestifer* strain WJ4 at 1 × 10^9^, 1 × 10^8^, 1 × 10^7^, 1 × 10^6^, 1 × 10^5^ CFU/mL) were prepared by 10-fold dilution with 0.01 M PBS solution (pH 7.4) to evaluate the sensitivity of the colloidal gold immunochromatographic strips. Samples of *R*. *anatipestifer* strains and other bacterial strains were diluted to 1 × 10^8^ CFU/mL with 0.01 M PBS solution (pH 7.4) to test colloidal gold immunochromatographic strip specificity. Stability assays were performed each month. Samples (100 μL) were added to the sample pad for detection and 0.01 M PBS solution (pH 7.4) was used as the blank control.

### Detecting samples

Sixty bacterial strains from clinical samples were grown in medium at 37ºC with aeration and suspended in 0.01 M PBS solution (pH 7.4). Samples (100 μL) were added to the sample pad. After 10 minutes, the color of test and control lines was noted. If both lines turned red, the sample was recorded as positive. If the control line turned red but the test line did not, the sample was considered negative. If neither line was colored, the strips were assumed to be nonfunctional.

## Results

### Characterization of the colloidal gold particle

**Fig 1:**
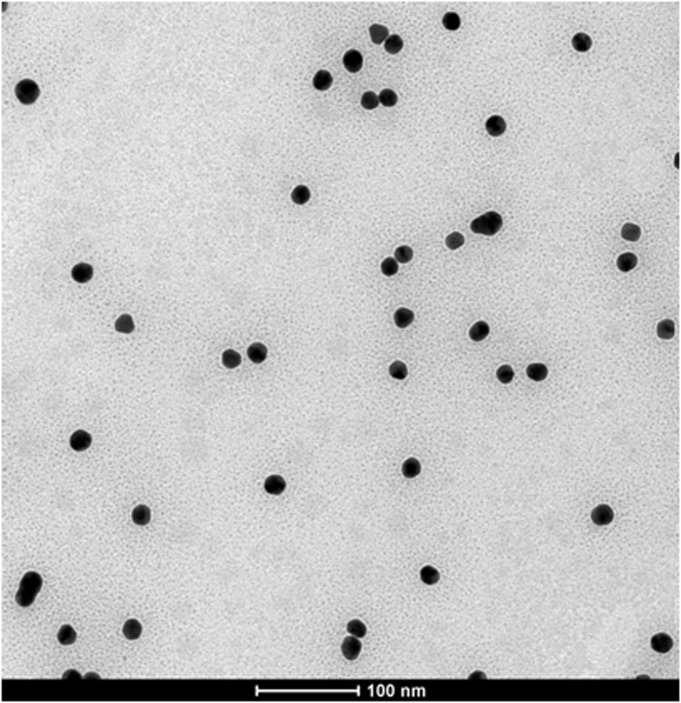
Transmission electron microscopy image of gold nanoparticles. Particles had varying sizes and shapes with an average diameter of 20±5.26 nm.

A chemical method of chloroauric acid reduction was chosen to synthesize colloidal gold particles. During colloidal gold preparation, the solution changed from colorless to wine red. During this process, gold ions were reduced to gold atoms in solution and the gold atoms immediately accumulated into colloidal gold. Colloidal gold particles were evaluated by TEM (Fig 1). The colloidal gold particles were spherical with an average diameter of 20±5.26 nm. No colloidal gold particle agglomeration was detected, suggesting that the colloidal gold particles were stable in solution, thus meeting the needs of colloidal gold for probes.

### Optimal conditions for conjugation and characterization of antibody-gold conjugates

The optimum pH of antibody adsorption for stable colloidal gold particles was determined to be 8.1. At this pH, a minimum of 3 μg/mL purified antibody was required to maintain stability. To ensure a sufficiently high antibody concentration stability and conjugation with gold particles, 5 μg/mL of purified antibody was used for the conjugation.

**Fig 2:**
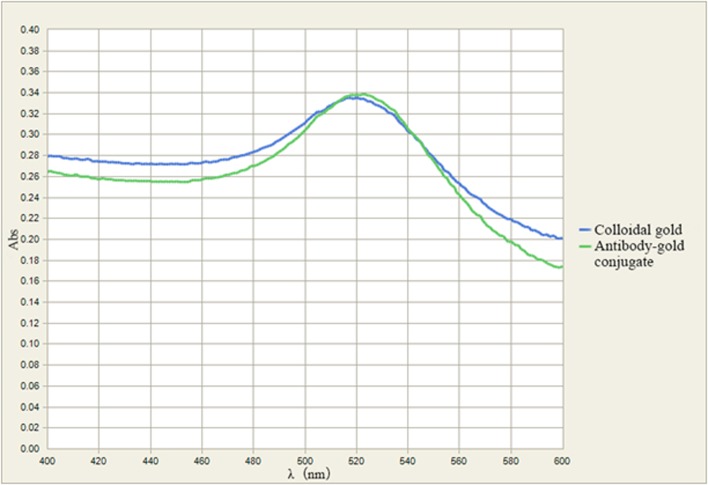
UV/Vis spectra of colloidal gold and the antibody-gold conjugate. Blue: colloidal gold solution; green: the antibody-colloidal gold conjugate.

In UV/Vis spectra of the colloidal gold and conjugates, peaks shifted when antibody was conjugated. Because of surface resonance of the colloidal gold particles, the colloidal gold curve peaked at 518 nm; when antibody was added, the surface resonance band shifted (Fig 2). A plasmon resonance band shift from 518 nm to 525 nm results from the interaction of antibody and colloidal gold particles [22–23].

### Specificity of the colloidal gold immunochromatographic strip

**Fig 3:**
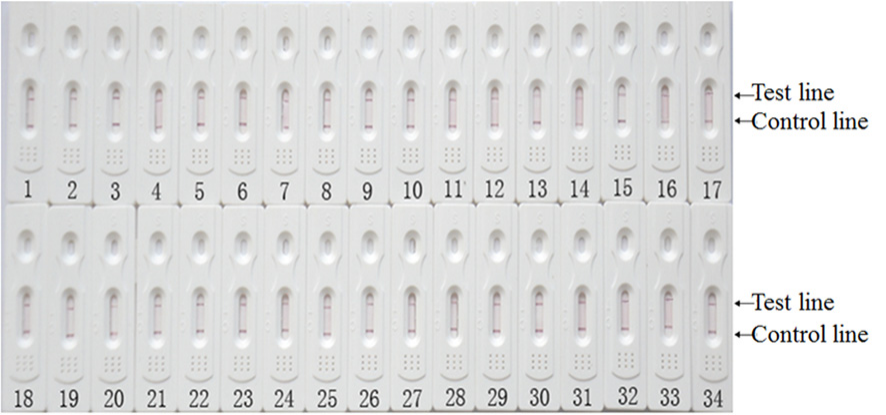
Colloidal gold immunochromatographic strips showed positive results for different serotypes of *R*. *anatipestifer* strains. 1–12: *R*. *anatipestifer* serotype 1 strains CH3, CH1, CQ1, CQ3, CQ4, CQ5, JY4, YXb12, NN2, NJ1, NJ4 and YL4 respectively; 13–23: *R*. *anatipestifer* serotype 2 strains JY1, SC2, NJ3, Yb2, Th4, YXb1, NN1, NN5, GD3, GD4 and GD5 respectively; 24: *R*. *anatipestifer* serotype 6 strain P2123; 25: *R*. *anatipestifer* serotype 8 strain D26220; 26–28: *R*. *anatipestifer* serotype 10 strains YXb11, HXb2 and YXL1; 29: *R*. *anatipestifer* serotype 12 strain 8785; 30–31: *R*. *anatipestifer* serotype 15 strains D743 and NN6; 32–33: Serotype not determined *R*. *anatipestifer* strains JY6 and GD6. 34: *R*. *anatipestifer* serotype 1 strain WJ4 (CGMCC 5264).

**Fig 4:**
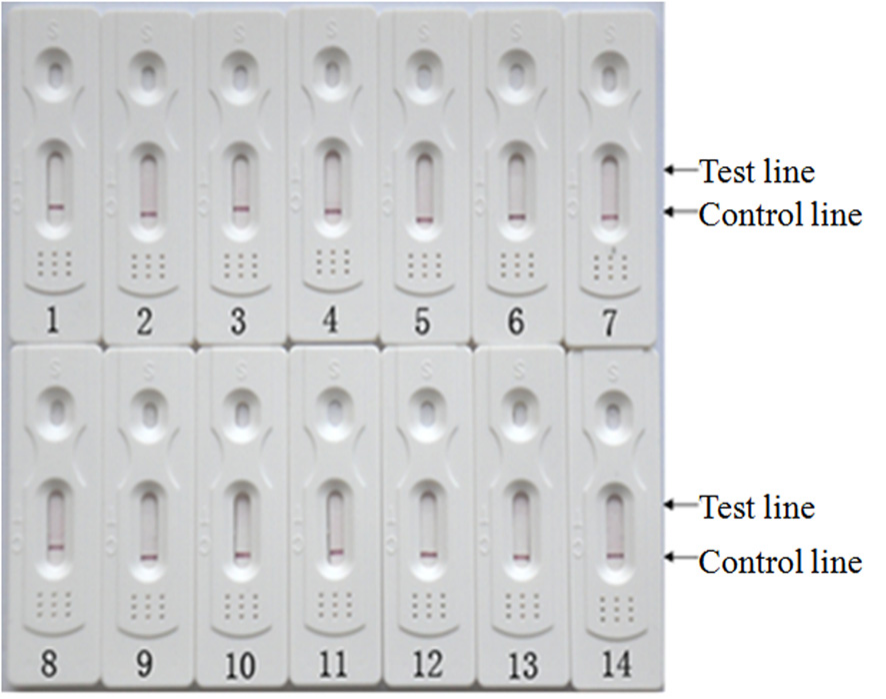
Colloidal gold immunochromatographic strips showed negative results for *Escherichia coli*, *Salmonella enterica* and *Pasteurella multocida*. 1: *E*. *coli* reference strain CVCC 1547; 2–3: *E*. *coli* serotype O1 strains APEC O1 and DE171; 4–5: *E*. *coli* serotype O2 strains DE14 and DE17; 6: *E*. *coli* serotype O18 strain CE66; 7–8: *E*. *coli* serotype O78 strains DE48 and DE56; 9–13: *S*. *enterica* strains CVCC1805, CAU0118, JXb1, CVCC 3384, and CVCC 519 respectively; 14: *P*. *multocida* strain CVCC 493.

Different *R*. *anatipestifer* strain serotypes and other bacterial species were used to evaluate the specificity of the colloidal gold immunochromatographic strips. Only samples containing *R*. *anatipestifer* showed two red lines in tests and control regions (Fig 3), while other samples showed a single red line at the control region (Fig 4). All 34 *R*. *anatipestifer* strains with different serotypes showed positive results with two red lines, indicating that the colloidal gold immunochromatographic strip had high reactivity and specificity for detecting *R*. *anatipestifer*. No cross-reaction was observed with other bacterial pathogen species.

### Sensitivity of the colloidal gold immunochromatographic strips

**Fig 5:**
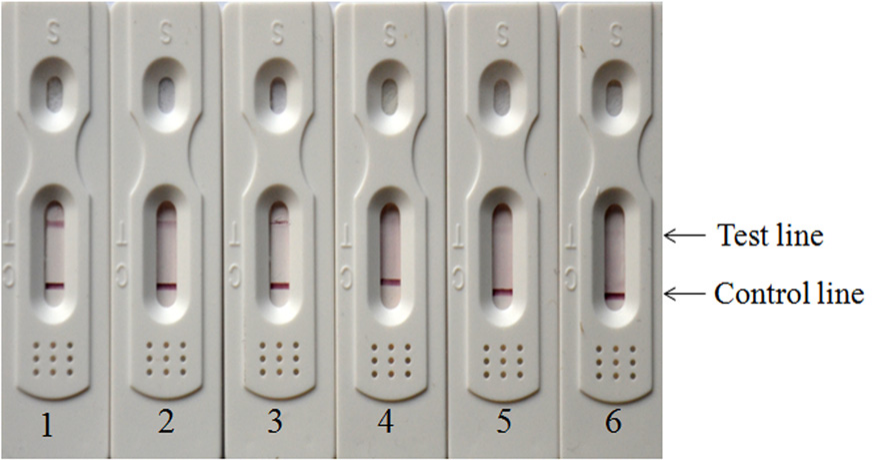
Sensitivity of colloidal gold immunochromatographic strips for detecting *R*. *anatipestifer* strain WJ4. 1–6, WJ4 at 10^8^, 10^7^, 10^6^, 10^5^, 10^4^, 10^3^ CFU/strip respectively.

To determine the sensitivity of colloidal gold immunochromatographic strip, different densities of *R*. *anatipestifer* strain WJ4 were prepared (Fig 5). When sample were diluted from 1 × 10^9^ CFU/mL to 1 × 10^6^ CFU/mL, two clear bands at the test and control lines were observed, while the low–density sample generated only a single band at the control line. Therefore, the sensitivity of the colloidal gold immunochromatographic strips was 1 × 10^6^ CFU of for *R*. *anatipestifer*. Similar results were observed in three repetitions of the samples (data not shown).

### Stability of the colloidal gold immunochromatographic strips

A batch of colloidal gold immunochromatographic strips was stored at 4°C for 6 months to evaluate the stability. Specificity and sensitivity for detection of *R*. *anatipestifer* were tested each month (Table 2). Colloidal gold immunochromatographic strips stored at 4°C for 6 months showed sensitivity that could detect 1 × 10^6^ CFU *R*. *anatipestifer*, the same as a freshly produced strip. Specificity for *R*. *anatipestifer* did not change. Therefore, the colloidal gold immunochromatographic strips could be stored at 4°C for at least 6 months without losses in sensitivity of specificity for *R*. *anatipestifer*.

**Table 2:** Specificity and sensitivity of colloidal gold immunochromatographic strip after 4°C storage.

Storage time (month)	Detection limit (CFU/strip)	Positive samples^a^	Negative samples^b^
0	10^6^	+	-
1	10^6^	+	-
2	10^6^	+	-
3	10^6^	+	-
4	10^6^	+	-
5	10^6^	+	-
6	10^6^	+	-

^a^ Simultaneously analyzed: 5 positive samples.

^b^ Simultaneously analyzed: 5 negative samples.

Experiments were repeated 3 times. +, Positive result; -, Negative result.

### Comparison of colloidal gold immunochromatographic strips to m-PCR and agglutination tests

Among the 60 clinical isolates, 34 were detected as *R*. *anatipestifer*-positive samples with two red lines by the colloidal gold immunochromatographic strips. The others were detected as *E*. *coli*-positive (18) and *S*. *enterica*-positive (8) samples using m-PCR. To compare colloidal gold immunochromatographic strips with m-PCR and agglutination tests, specificity, sensitivity and detection time were investigated (Table 3). Using positive samples, 100% (34/34) were detected by the colloidal gold immunochromatographic strips and m-PCR assays. However, 88.24% (30/34) of positive samples were detected using agglutination tests, suggesting that colloidal gold immunochromatographic strips had a high accuracy for detecting *R*. *anatipestifer*. Colloidal gold immunochromatographic strips had 500 times higher sensitivity than conventional agglutination test and 20 times lower sensitivity than m-PCR. Colloidal gold immunochromatographic strip results were read within 10 min, which was less time than for m-PCR results.

**Table 3:** Comparison of colloidal gold immunochromatographic strips, m-PCR and agglutination test for detecting *Riemerella anatipestifer*.

Methods	Specificity		Detection limit (CFU/strip)	Detection Time (min)
	positive/total	negative/total		
Strip^a^	34/34	14/14	1×10^6^	10
m-PCR	34/34	14/14	5×10^4^	150
Agglutination Test	30/34	14/14	5×10^8^	3

^a^ Colloidal gold immunochromatographic strip.

## Discussion

*R*. *anatipestifer* infection is an important bacterial infectious disease of avian species worldwide. Currently, several methods are available for detecting of *R*. *anatipestifer*. Serological methods, such as AGID and ELISA [5] are labor-intensive and time-consuming, making them unsuitable for rapid and simple characterization of *R*. *anatipestifer* infections. PCR [7], m-PCR [8] and LAMP [9] detect *R*. *anatipestifer* with high sensitivity. However, these methods usually require special primers, skilled technicians and particular equipments, which also make them difficult for the rapid and simple characterization of *R*. *anatipestifer* infection. In comparison, the colloidal gold immunochromatographic strips we developed were easy to operate and could save time and money because they did not require skilled technicians or special equipment and the results were visually determined directly within 10 min. The sensitivity of the colloidal gold immunochromatographic strips was 500 times higher than a conventional agglutination test. Compared to m-PCR method, the colloidal gold immunochromatographic strips were faster and simpler with results read within 10 min. Although m-PCR method is more sensitive than our method, it requires expensive instruments and specific laboratory skills and takes 2.5 h to complete. Consequently, the colloidal gold immunochromatographic strips developed in this study have advantages for the rapid detection of *R*. *anatipestifer*.

The specificity and sensitivity of the colloidal gold immunochromatographic strips display a substantial correlation with the following factors. First, the quality of the MAb used in the strip test is crucial for the specificity and sensitivity. Our previous study showed that *R*. *anatipestifer* GroEL shares more than 98% similarity among *R*. *anatipestifer* strains. Moreover, *R*. *anatipestifer* GroEL is antigenic and protective [24]. In this study, GroEL was used as the target in the colloidal gold immunochromatographic strips for detecting *R*. *anatipestifer*. In our previous study, two stable hybridoma cell clones (1G2B6, 1G2F10) producing anti-GroEL MAbs were established. Both MAbs specifically recognized *R*. *anatipestifer* and had high titers of their antigen epitopes by Western blots and ELISAs. In analysis of the colloidal gold immunochromatographic strip assays, only MAb 1G2F10 specifically detected the *R*. *anatipestifer* strains. Second, pretreatment of the sample pad and conjugate pad were crucial to pick up the release speed of the conjugate and reduce the non-specificity. Several factors could be optimized such as surfactant ratio and blocking solution. In this study, we used different concentrations of Tween-20, polyvinylpyrrolidone K30 and sodium azide, and found that 0.01 M PBS containing 2% BSA, 0.5% sucrose, 0.5% Tween-20, 0.5% polyvinylpyrrolidone K30, and 0.02% sodium azide (pH 7.4) was the most suitable blocking solution. The type of nitrocellulose membrane is critical for a highly specific, sensitive, and rapid colloidal gold immunochromatographic strip assay. The key characteristics for membrane suitability are wicking rate and speed of liquid diffusion on the membrane [25]. Several types of strips have been developed for rapid colloidal gold immunochromatographic detection of pathogens, using nitrocellulose, polyethylene, polyethersulfone, and nylon or fused silica-based membrane [26–28]. Although nitrocellulose membranes are the most commonly used, their capillary flow properties might make a huge difference due to the physical and chemical nature of the membrane material [29–30]. The capillary flow properties, in turn, affect assay specificity and sensitivity, and test line consistency [29]. Membranes with a larger pore size accelerate detection test rates through lower wicking and higher diffusion rates. However, membrane pore size and protein-binding capacity negatively correlate. Thus, this kind of membrane usually has a lower protein-binding capacity, potentially leading to low sensitivity. We found that 150-s/4-cm nitrocellulose membranes were optimal for our system through a series of tests.

In summary, we developed highly specific and sensitive colloidal gold immunochromatographic strips for detecting *R*. *anatipestifer*. The colloidal gold immunochromatographic strip assay could be a practical tool for screening large numbers of samples during outbreaks. This assay offers a rapid method for differentially diagnosing common poultry bacterial diseases, such as *E*. *coli*, *S*. *enterica* and *P*. *multocida*. Thus, the colloidal gold immunochromatographic trips could be used for epidemiology study and disease control of *R*. *anatipestifer* infections.
